# Assessing TB treatment outcomes in pregnant women living with HIV with drug-susceptible TB

**DOI:** 10.5588/pha.24.0051

**Published:** 2025-03-01

**Authors:** N. Hernandez-Morfin, S. Cohn, Z. Waja, R.E. Chaisson, N. Martinson, N. Salazar-Austin

**Affiliations:** ^1^ Department of Pediatrics, Johns Hopkins University School of Medicine, Baltimore, MD, USA;; ^2^ Department of Medicine, Johns Hopkins University School of Medicine, Baltimore, MD, USA;; ^3^ Perinatal HIV Research Unit, University of the Witwatersrand, Johannesburg, South Africa.

**Keywords:** pregnancy, maternal health, tuberculosis, TB-HIV co-infection, TB, treatment outcomes

## Abstract

**BACKGROUND:**

Limited data exist on TB treatment outcomes among pregnant women with TB. Physiological and immunological adaptations during pregnancy may affect the efficacy of TB treatment. We aimed to evaluate factors associated with unsuccessful TB treatment outcomes among pregnant women living with HIV (PWLHIV) and diagnosed with TB in the Tshepiso study.

**METHODS:**

In this secondary analysis, we used multivariable logistic regression to evaluate factors associated with unsuccessful TB treatment outcomes among PWLHIV with drug-sensitive TB disease enrolled in the Tshepiso study in Soweto, South Africa, from 2011–2014.

**RESULTS:**

This analysis includes 79 PWLHIV diagnosed with drug-sensitive TB during pregnancy; 18 (23%) had an unsuccessful treatment outcome. Factors associated with unsuccessful TB treatment include detectable HIV RNA viral load at enrollment to the study (aOR 5.1, 95% CI 1.1–25.3), presence of extrapulmonary TB (aOR 2.2, 95% CI 0.4–11.7), bacteriological (positive smear and/or culture) confirmation of TB (aOR 2.1, 95% CI 0.7–6.7), and anemia (Hb ≤ 10.5 g/dL) (aOR 1.0, 95% CI 0.3–3.1). The only factor with statistical significance was a detectable HIV RNA viral load.

**CONCLUSION:**

Detectable HIV viral load emerges as a critical factor associated with an unsuccessful TB treatment outcome in pregnant women living with HIV and diagnosed with TB.

TB continues to be a leading cause of death among women of childbearing age globally.^1,2^ An estimated 216,000 pregnant women develop TB each year, and in sub-Saharan Africa, these women bear a disproportionately high rate of HIV coinfection.^3^ Pregnant women with TB have an increased risk of maternal and infant mortality, prematurity and low birth weight, and among those mothers living with HIV, an increased risk of perinatal HIV transmission.^4,5^

Immunological changes during pregnancy may affect the susceptibility to and severity of infections and could result in the altered success of TB treatment.^6^ Additionally, TB drug concentrations may be reduced in pregnant women due to volume changes, and the clinical impact on TB treatment outcomes is unknown.^7^ In the general population, factors associated with unsuccessful TB treatment outcomes (treatment failure, death and loss to follow-up) include older age, male sex, underweight, positive pulmonary smear, extrapulmonary TB, long distance from the nearest health facility, and lack of family support.^8–12^

Two observational studies of pregnant women with drug-sensitive TB in South Africa (*n* = 76) and Peru (*n* = 36) found different rates of unsuccessful TB treatment outcomes, 45% and 2.4%, respectively.^13,14^ In South Africa, poor outcomes were largely driven by loss to follow-up. Factors associated with an unsuccessful TB treatment outcome included HIV coinfection, extrapulmonary TB, and having a low-birth-weight infant. A meta-analysis of TB treatment outcomes among 275 pregnant women with drug-resistant TB from 10 studies showed high rates of treatment success, similar to non-pregnant populations.^15^ Small sample sizes and variability in reported outcomes limit our understanding of TB treatment outcomes among pregnant women.

Due to the limited information on TB treatment outcomes in pregnant women, we performed a secondary analysis of the Tshepiso study. Specifically, we aim to evaluate factors associated with unsuccessful treatment outcomes among pregnant women with HIV and diagnosed with drug-susceptible TB (DS-TB).

## METHODS

Tshepiso was a prospective matched cohort study of pregnant women living with HIV with and without TB disease.^16^ The study was designed to study the impact of TB disease on maternal, pregnancy and infant outcomes in pregnant women living with HIV. For this purpose, Tshepiso enrolled pregnant women aged ≥18 years who were at least 13 weeks of gestation from 10 prenatal clinics in Soweto, South Africa, between 2011 and 2014. Pregnant women were clinically diagnosed with TB, with or without bacteriologic confirmation, during all stages of pregnancy. The study was noninterventional; all women received care as per South African antiretroviral therapy (ART) and TB guidelines.^17–19^ Pregnant women were followed during the second and third trimesters of pregnancy, and mother-infant pairs were followed within 1 week of delivery and at 6 weeks, 6 months, and 12 months post-delivery.

In this secondary analysis, we analyzed the Tshepiso cohort data to evaluate the TB treatment outcomes among the cohort’s study participants.

### Prevention and treatment guidelines for HIV and TB

DS-TB treatment was 2 months of daily rifampicin, isoniazid, pyrazinamide, and ethambutol followed by 4 months of daily rifampicin and isoniazid, with standard weight-based dosing. HIV treatment consisted of either efavirenz-based ART (CD4 ≤ 350 cells/mm³) or zidovudine (AZT) monotherapy (CD4 > 350 cells/mm³) with single-dose nevirapine at the initiation of labor, intrapartum zidovudine, and one postpartum dose of tenofovir and emtricitabine until 2013, when South Africa began to provide ART to all pregnant women with HIV, irrespective of CD4 count.^16–19^

### Exposures and outcomes

Demographic characteristics (age, race, education and income) and pregnancy history were self-reported at enrollment. The gestational age of the participants was determined by the first day of the last menstrual period. Weight, height, body mass index (BMI), hemoglobin, CD4, HIV RNA viral load, smear and culture were measured at enrollment. Bacteriological confirmation of TB disease (smear and/or culture) was done at enrollment. However, because enrollment did not necessarily coincide with the initiation of TB treatment, baseline smear and culture data are not available for all participants.

TB treatment, maternal and infant outcomes were confirmed from the mother’s TB, maternity, and hospital records and the infant’s road to health card. For this analysis, anemia was defined as a hemoglobin level of <10.5 g/dL, in accordance with the WHO criteria for the second and third trimesters of pregnancy, to distinguish normal physiologic changes of pregnancy from clinically significant anemia. We categorized extrapulmonary TB as active TB outside the lungs,^20^ bacteriological confirmation as having a positive acid-fast bacilli smear and/or culture, and a detectable viral load as 20 or more copies/mL.

Treatment outcomes were classified per 2021 WHO definitions.^21^ We utilized the sustained treatment success outcome definition for operational research, defined by individuals who were cured or completed TB treatment and were assessed at 6 months after treatment completion, who remain alive and free of TB. For shorter writing purposes, we will refer to them as ‘successful’ TB treatment outcomes in this paper. Unsuccessful TB treatment outcomes include treatment failure, death and loss to follow-up.

### Statistical analysis

The pregnant woman’s demographic and clinical characteristics were described using proportions for categorical variables and mean with standard deviations (SDs) and medians with interquartile ranges (IQRs) for normally and nonnormally distributed continuous variables, respectively. We used the Pearson χ^2^ test to compare categorical variables and the Student *t*-test to compare continuous variables. Multivariable logistic regression modeling was performed to describe the association between identified risk factors and unsuccessful TB treatment outcomes. The selection of covariates for the multivariable analysis was based on previous risk factors for poor treatment outcomes identified in the literature review. A variance inflation factor (VIF) analysis was conducted to assess collinearity among the variables. The data were analyzed using Stata statistical software v14 (Stata Corp, College Station, TX, USA).^22^

### Ethical approval

Institutional review boards at the Johns Hopkins Medicine (Baltimore, MD, USA) and the University of Witwatersrand (Johannesburg, South Africa) approved the study. Written informed consent was provided by all participants prior to enrollment.

## RESULTS

The Tshepiso study enrolled 80 pregnant women living with HIV and TB disease. In our secondary data analysis, we excluded 1 participant who had confirmed drug-resistant TB (DR-TB), leaving 79 participants with DS-TB.

Unsuccessful treatment outcome was identified in 18 (23%) women, including 12 participants with treatment failure, 1 participant who died less than 42 days after delivery, and 5 participants who were lost to study follow-up (Figure). Among the 61 participants with a successful treatment outcome, two were classified as cured because of the presence of a negative bacteriological sample at the end of treatment, and 59 participants had treatment completion. A second death occurred in a pregnant woman 7 months after completing TB treatment. Given that the death occurred more than 6 months after completing TB treatment, she was classified as having completed treatment. The cause of death was a hepatic failure and was not thought to be related to TB treatment.

**FIGURE.**
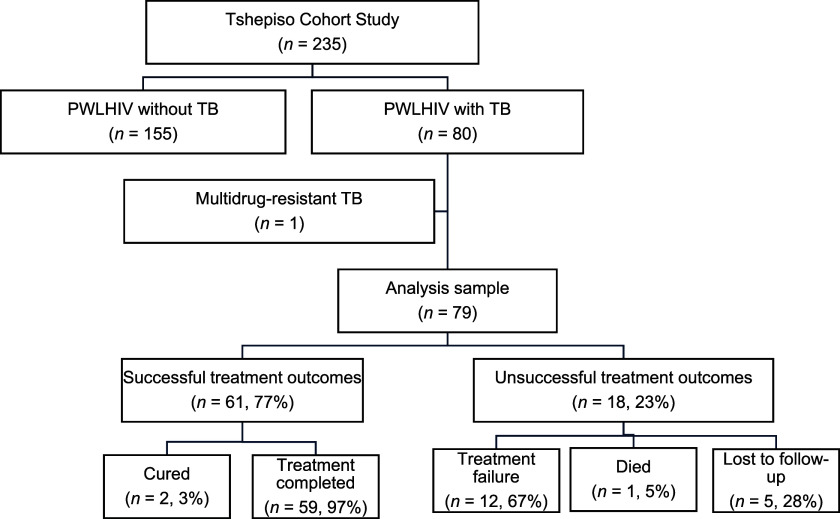
Participant flow diagram for inclusion in the study analysis. PWLHIV = pregnant women living with HIV.

The study participants had similar characteristics at baseline with a median maternal age of 29 years (interquartile range [IQR] 26–32) in the successful TB treatment outcome group and 28 years (IQR 24–34) in the unsuccessful TB treatment outcome group (*P* = 0.70). The median gestational age at enrollment was 29 weeks (IQR 26–34) and 30 weeks (IQR 25–33), respectively (*P* = 0.646). The median gestational age at TB diagnosis was 24 weeks vs 29 weeks, respectively (*P* = 0.448). They also had similar parity, BMI, socio-economic and hemoglobin values at enrollment (Table 1).

**TABLE 1.** Characteristics of pregnant women at enrollment.

Enrollment characteristics	Successful treatment outcomes	Unsuccessful treatment outcomes	
	(*n* = 61)	(*n* = 18)	
	*n* (%)	*n* (%)	*P*-value univariate analysis
Age, years, median [IQR]	29 [26–32]	28 [24–34]	0.696
Gestational age, weeks, median [IQR]	29 [26–34]	30 [25–33]	0.646
Number of previous deliveries:			0.305
0	3 (6)	1 (8)	
1	27 (50)	7 (54)	
2	18 (33)	2 (15)	
≥3	6 (11)	3 (23)	
Height, cm, median [IQR]	160 [154–165]	160 [158–161]	0.673
Weight, kg, median [IQR]	62 [53 –70]	57 [56–63]	0.242
Body mass index, kg/m^2^, median [IQR]	24.6 [19.0–32.3]	23.2 [21.7–25.5]	0.300
Education			0.927
8^th^ grade or below	12 (20)	3 (17)	
9^th^–12^th^ grade	26 (42)	9 (50)	
Completed high school	20 (33)	5 (27)	
Started or completed tertiary degree	3 (5)	1 (6)	
Hemoglobin, g/dL, median [IQR]	10.6 [9.8–11.9]	10.5 [10–11.4]	0.363
CD4 cell count, cells/uL, median, [IQR]	288 [156–385]	144 [88–304]	0.161
Category CD4 count, cells/uL			0.141
<100	8 (13)	6 (33)	
<350	32 (53)	8 (44)	
350–499	13 (21)	1 (6)	
≥500	8 (13)	3 (17)	
HIV RNA, ≥20 copies/mL (detectable)	38 (62)	16 (89)	0.033*
HIV RNA, copies/mL			0.111
<20 (undetectable)	21 (35)	2 (11)	
20–999	17 (28)	6 (33)	
1,000–100,000	19 (31)	7 (39)	
>100,000	2 (3)	3 (17)	
N/A	2 (3)	0 (0)	
ART at enrollment			0.794
AZT monotherapy	17 (28)	6 (33)	
Combination ART	41 (67)	12 (67)	
None	3 (5)	0 (0)	
ART at delivery			0.01*
AZT monotherapy	7 (12)	1 (6)	
Combination ART	52 (85)	14 (77)	
None	2 (3)	3 (17)	
Past TB	14 (23)	6 (33)	0.373
TB site			0.295
Pulmonary	56 (92)	15 (83)	
Extrapulmonary	5 (8)	3 (17)	
Extrapulmonary location			0.158
Disseminated	1 (1.6)	0 (0)	
Lymph node	2 (3.3)	0 (0)	
Pericardial	1 (1.6)	0 (0)	
Pleural	1 (1.6)	2 (12)	
Pulmonary/abdomen	0 (0)	1 (6)	
Bacteriological confirmation positive	31 (51)	11 (61)	0.442
Time to culture positivity, days, median [IQR]	25 [17–29]	21 [18–25]	0.317
Weeks of pregnancy TB diagnosis, median [IQR]	24 [19–30]	29 [23–32]	0.448
Pregnancy trimester when TB treatment started			0.080
Before pregnancy	4 (7)	1 (6)	
First trimester	3 (5)	1 (6)	
Second trimester	30 (49)	3 (16)	
Third trimester	24 (39)	13 (72)	

* Statistically significant.

IQR = interquartile range; ART = antiretroviral therapy; AZT = zidovudine.

The median CD4 cell count was 288 (IQR 156–385) and 144 (IQR 88–304) in the successful and unsuccessful treatment outcome groups, respectively (*P* = 0.161); 38% of participants with successful TB treatment outcomes had an undetectable RNA HIV viral load (<20 copies/ml) compared to 11% with unsuccessful TB treatment outcomes (*P* = 0.033).

At enrollment, 67% of participants in both groups were on combination ART. By the time of delivery, 85% of participants who completed TB treatment were on combination ART compared with 77% of participants with unsuccessful TB treatment outcomes (*P* = –0.01).

The two groups had similar proportions patients reportinga previous TB episode (23% vs 33%, respectively; *P* = 0.373). Most participants had pulmonary TB (92% vs 83%, respectively; *P* = 0.295).

Over half of each group had bacteriological confirmation (51% vs 61%, respectively; *P* = 0.442). Median days to culture positivity were similar, 25 vs 21 days, respectively (*P* = 0.317).

For the logistic regression model, we included the following variables: having an extrapulmonary TB diagnosis, anemia, bacteriologic confirmation of disease, and detectable HIV RNA viral load (≥20 copies/ml). No significant collinearity was found between these variables (all VIF values <2). Participants with extrapulmonary disease had 2.2 times higher odds (adjusted odds ratio [aOR] 2.2, 95% CI 0.4–11.7; *P* = 0.352) of unsuccessful TB treatment outcomes compared to women with pulmonary TB (Table 2). Participants with bacteriologic confirmation had 2.1 times higher odds (aOR 2.1, 95% CI 0.7–6.7; *P* = 0.211) of unsuccessful TB treatment outcomes compared to women without bacteriologic confirmation. Anemia was not associated with the outcome (aOR 1.0, 95%CI 0.3–3.1). Detectable HIV viral load at enrollment was associated with an unsuccessful TB treatment outcome (aOR 5.1, 95% CI 1.1–25.3; *P* = 0.045).

**TABLE 2.** Unadjusted and adjusted risk of unsuccessful TB treatment outcomes.

Characteristic	Unadjusted OR		Adjusted OR	
	OR (95% CI)	*P*-value	OR (95% CI)	*P*-value
Extrapulmonary TB	2.2 (0.5–10.5)	0.305	2.2 (0.4–11.7)	0.352
Anemia (Hg <10.5 g/dL)	1.3 (0.4–3.6)	0.668	1.0 (0.3–3.1)	0.993
Bacteriologic confirmation (smear and/or culture)	1.5 (0.5–4.4)	0.444	2.1 (0.7–6.7)	0.211
Detectable viral load (≥20 copies/mL)	4.8 (1.1– 23.0)	0.047*	5.1 (1.1–25.3)	0.045*

* Statistically significant.

OR = odds ratio; CI = confidence interval; Hg = hemoglobin.

## DISCUSSION

The main finding from our study is that 77% of pregnant women with TB and HIV had successful TB treatment outcomes. This is lower compared to the global successful rate among people with DS-TB of 85–86%, but it is similar to the global estimate of successful TB treatment outcomes among people living with HIV and DS-TB.^2,20^ Previous studies in pregnant populations with drug-susceptible TB found varying treatment success rates of 55% to 96%. In the South African cohort study with a success rate of 55% and a high proportion of HIV coinfection, unsuccessful outcomes were largely driven by loss to follow-up, where few women experienced treatment failure or death.^14^ In a Peruvian cohort with a low incidence of HIV coinfection, 96% of pregnant women with TB had successful TB treatment outcomes.^13^ It is possible that unsuccessful treatment outcomes are driven by TB/HIV coinfection. As the study was conducted only among people living with HIV, HIV-coinfection itself could not be assessed as a risk factor for unsuccessful TB treatment outcomes.^13,14^ The added value of this cohort study is that we have relatively high retention and associations with treatment outcomes largely reflect the association with treatment failure and death.

This study also explored specific factors that could identify patients at higher risk of an unsuccessful treatment outcome. Detectable HIV viral load at enrollment was associated with unsuccessful TB treatment outcomes, highlighting the importance of viral control and ART adherence during TB treatment. The wide confidence interval reflects imprecision, likely due to our small sample size. Despite this limitation, the association aligns with the biological plausibility of HIV control impacting TB treatment efficacy.

Extrapulmonary TB and bacteriologic confirmation of TB disease have both been previously identified as factors associated with unsuccessful treatment outcomes, similar to the results in this study.^23^ Although smear status may be considered a better measure of disease severity at baseline, smear results were not known at diagnosis for all participants. We, therefore, combined smear and culture status as a marker of disease confirmation rather than disease severity.

Decreased hemoglobin concentration may be an indicator of chronic disease and/or poor nutrition. Anemia has been described as a marker of poor health at treatment initiation and is associated with mortality and unsuccessful TB treatment outcomes in adults.^10,24^ In our study, there was no association between anemia and treatment outcomes. The cutoff for anemia was defined by WHO as less than 10.5 g/dL during the second trimester of pregnancy, which differs from some other studies (12.0 mg/dl), which may have influenced our results.^24^

### Limitations

Pregnant women with TB disease are challenging to recruit and enroll on research studies. The total sample size of 79 pregnant women with TB disease is small, thereby limiting the precision of associations between study characteristics and unsuccessful treatment outcomes. In the future, meta-analyses for TB treatment outcomes among pregnant women with DS-TB may enhance our understanding of DS-TB treatment outcomes in this important population.

Pregnant women were not necessarily enrolled at TB diagnosis, limiting our ability to assess disease severity at presentation, including smear status, BMI and anemia. Because women were already pregnant at enrollment, we were unable to obtain data on pre-pregnancy weight to assess their nutritional status. Therefore, we could not examine the association between nutritional status and TB treatment outcomes during pregnancy despite poor nutrition being associated with unsuccessful TB treatment outcomes.^25–27^

## CONCLUSION

Pregnant women with TB-HIV can achieve successful treatment outcomes similar to global estimates. Detectable HIV RNA viral load measured at the start of or during TB treatment was associated with an unsuccessful TB treatment outcome. Programs should target this population with enhanced follow-up to not only prevent mother-to-child transmission of HIV but also to improve TB treatment outcomes.
